# Supplementary material for: Clinicopathological Factors Associated with Oncotype DX Risk Group in Patients with ER+/HER2- Breast Cancer
Source: Cancers (Basel). 2023 Sep 7;15(18):4451. doi: 10.3390/cancers15184451 (PMC10527468; doi:10.3390/cancers15184451)
Supplement: Supplementary file 1 [file cancers-15-04451-s001.zip › cancers-2574213-supplementary.pdf]

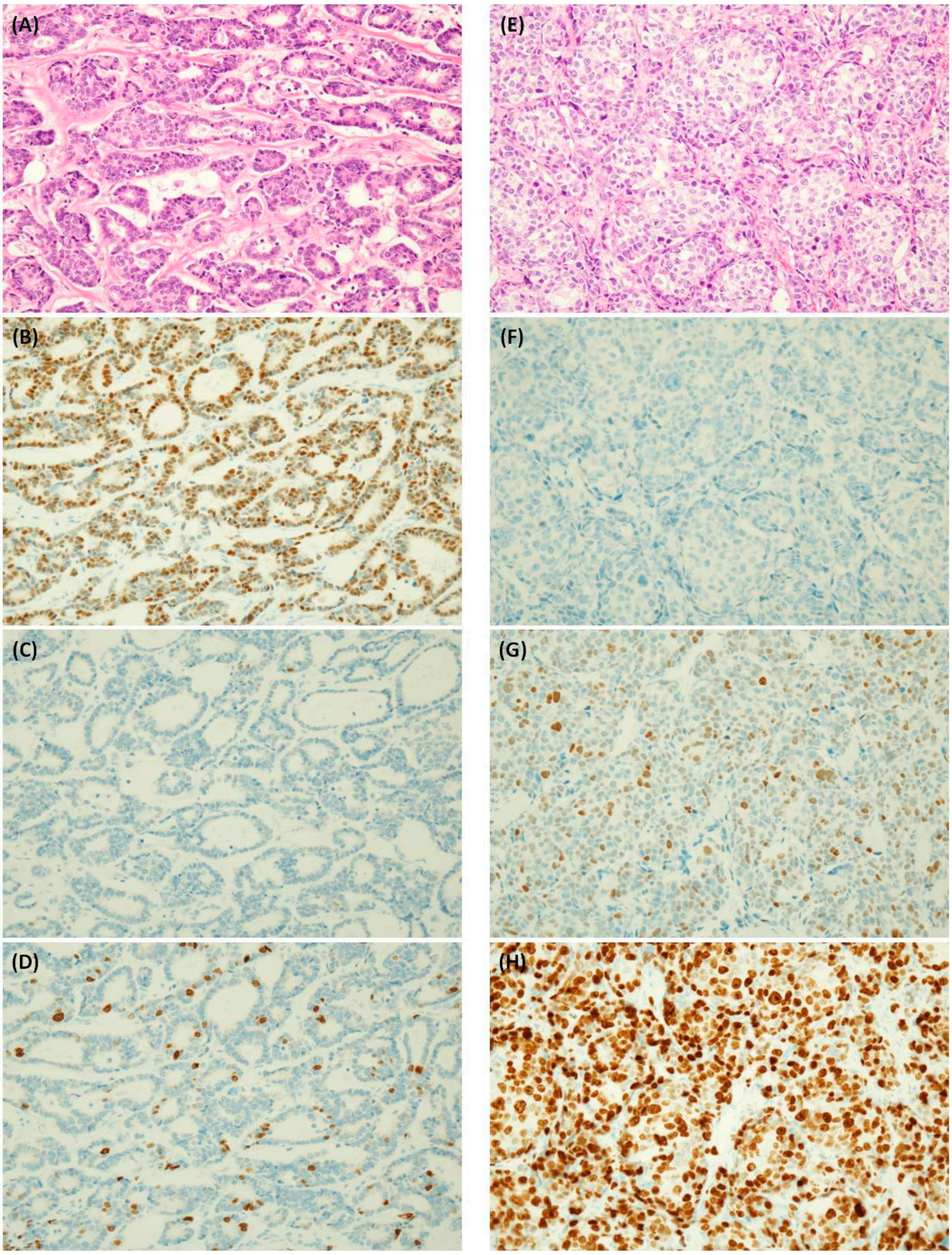

Figure S1. Comparison of pathological findings between the low-risk and high-risk groups. The representative tumor in the low-risk group shows (A) histologic grade 1, (B) strong positivity for progesterone receptor, (C) immuno-negativity for p53, and (D) a low Ki-67 labeling index, whereas the representative tumor in the high-risk group shows (E) histologic grade 3, (F) weak positivity for progesterone receptor, (G) immuno-reactivity for p53, and (H) a high Ki-67 labeling index. (A & E: H&E, x20; B, C, D, F, G & H: IHC, x20)

H&E, Hematoxylin and Eosin staining; IHC, immunohistochemical staining
